# Ice thickness and volume changes across the Southern Alps, New Zealand, from the little ice age to present

**DOI:** 10.1038/s41598-020-70276-8

**Published:** 2020-08-07

**Authors:** Jonathan L. Carrivick, William H. M. James, Michael Grimes, Jenna L. Sutherland, Andrew M. Lorrey

**Affiliations:** 1 School of Geography and water@leeds, University of Leeds, Woodhouse Lane, Leeds, LS2 9JT West Yorkshire UK; 2 National Institute of Water and Atmospheric Research, Auckland, New Zealand

**Keywords:** Cryospheric science, Climate-change impacts

## Abstract

Rapid changes observed today in mountain glaciers need to be put into a longer-term context to understand global sea-level contributions, regional climate-glacier systems and local landscape evolution. In this study we determined volume changes for 400 mountain glaciers across the Southern Alps, New Zealand for three time periods; pre-industrial “Little Ice Age (LIA)” to 1978, 1978 to 2009 and 2009 to 2019. At least 60 km^3^ ± 12 km^3^ or between 41 and 62% of the LIA total ice volume has been lost. The rate of mass loss has nearly doubled from − 0.4 m w.e year^−1^ during 1,600 to 1978 to − 0.7 m w.e year^−1^ at present. In comparison Patagonia has lost just 11% of it’s LIA volume. Glacier ice in the Southern Alps has become restricted to higher elevations and to large debris-covered ablation tongues terminating in lakes. The accelerating rate of ice loss reflects regional-specific climate conditions and suggests that peak glacial meltwater production is imminent if not already passed, which has profound implications for water resources and riverine habitats.

## Introduction

Mountain glaciers and ice caps produce global sea level rise contributions that over the last few decades have far exceeded those from the major ice sheets^1,2^. With decadal mean contributions of ~ 0.9 mm year^−1^ they account for 25% of total global sea level rise^3^. Furthermore, those contributions have accelerated recently^4,5^ and are expected to continue for many decades^6^. It is therefore crucial to understand not only the present day distribution of ice volume and contemporary rates of decline, but also to place modern rates of cryosphere and proglacial landscape change within a longer-term context. Reconstructing mountain glacier volumes is perhaps most obviously achieved by using moraines and trimlines that delimit former ice margin extent and ice thickness^7^.

Few studies have sought to quantify glacier volume changes in the Southern Hemisphere on a centennial-scale; i.e. since recession from nineteenth Century maximum ice limits, or the conclusion of pre-industrial times that is contemporaneous with the “Little Ice Age (LIA)” end. Some exceptions include the geomorphology-based studies by Glasser et al.^8^ for the Patagonian ice fields, Carrivick et al.^9^ for several mountain glaciers on James Ross Island on the Antarctic Peninsula and that by Mark et al.^10^ for a small number of glaciers around Quelccaya ice cap in northern Peru. In a different approach using a parameterised mass balance gradient and an estimate of ice thickness applied along single glacier flow lines, Hoelzle et al.^11^ have calculated an ice volume loss across the Southern Alps of New Zealand of − 61% since the LIA. The paucity of distributed ice volume reconstructions, and hence the scant knowledge on the response of Southern Hemisphere glaciers to climate change since late pre-industrial times, is especially important to understand because there are inter-hemispheric differences in Holocene climate change (e.g.^12-16^). In addition, spatiotemporal heterogeneity for climate variability within the Southern Hemisphere is strong (e.g.^17,18^), and differences within regions can be amplified due to modes of variability that drive synoptic weather patterns^19–23^.

There have been limited estimates of ice volume loss across the Southern Alps of New Zealand during the late Holocene^24^. This knowledge gap is important because those glaciers are highly responsive to climate change, especially air temperature changes^25^. Those short response times, coupled with high sediment availability, has produced exceptionally well-preserved sets of moraines, which delineate former ice margins (e.g.^26^). The most widespread and best-preserved moraines and trimlines across the Southern Alps represent the former extent of mountain glaciers between the fourteenth and nineteenth centuries, which is contemporaneous with the northern hemisphere LIA^15,27^. Details about the local expression of late pre-industrial era Southern Alps moraines that overlap with the classical definitions of northern European LIA are described in our Table SI 1. In this study we adopt the term LIA for simplicity to indicate that we have targeted the most recent multi-centennial expression of greatly expanded glaciers that have since retreated.

Determination of modern rates of glacier geometry changes are complicated in New Zealand by limited direct and remotely sensed information, overall ice terminus advances during the 1980s and 1990s^25^, and highly variable glacier responses to climate depending on terminus type (including proglacial calving in lakes), glacier hypsometry and glacier surface conditions^28,29^. In particular, the largest glaciers are presently stagnating and down-wasting (e.g.^30–32^) which distorts the apparent loss of ice if that estimate is based solely on areal extent. The aim of this study is therefore to quantify glacier volume loss since the “Little Ice Age” for the Southern Alps.

## Datasets and methods

Glacier outlines for the year 1978 by Chinn^33^ for the entire Southern Alps were obtained from the Randolph Glacier Inventory (RGI) via Global Land Ice Measurements from Space (GLIMS)^34^. We acknowledge the imperfections of this dataset but did not seek to subjectively modify it. Glacier outlines for year 2009 mapped by Sirguey and More^35^ for the Mt Cook region only were also obtained from GLIMS. In this study we derived glacier outlines for year 2019 using a land cover classification of 10 m resolution multi-spectral Sentinel-2 level-2A satellite imagery. The Sentinel-2 spectrally derived bands included the NDVI, NDSI, NDWI and the first principal component of an eigenvector analysis on a grey-level co-occurrence matrix analysis. The classification was conducted in Google Earth Engine using a Random Forest machine learning classifier (n = 50). Supplementary information (SI) has further detail on image pre-processing, image band calculation and the classification method.

**Figure 1:**
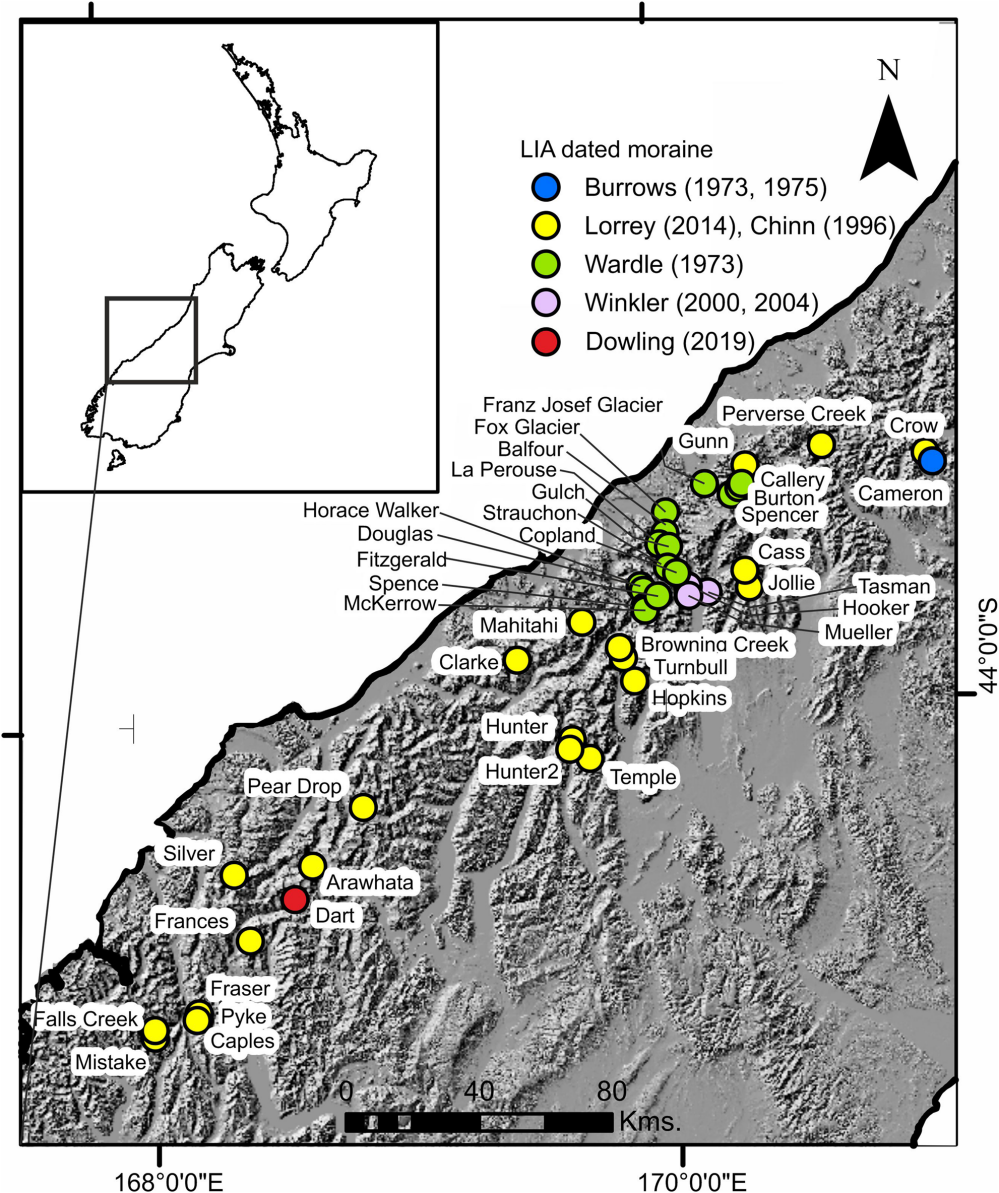
Sites across the Southern Alps, New Zealand where glacier moraines have been dated to the LIA by several authors. Dates attributed to the LIA are detailed in Table SI 1.

Glacier outlines for the LIA were primarily mapped using a hillshade of an 8 m Digital Elevation Model (DEM)^36^. First, crests of LIA-associated moraines^37^ were mapped (Fig. 1), and those outlines were extended to incorporate associated trimlines. Where multiple moraine crests were present, the innermost was chosen to provide a conservative estimate of LIA area. Next, the three largest glaciers (Tasman Glacier, Mueller Glacier, Hooker Glacier) located near Mt Cook village^14,38–40^ and the few other LIA-dated moraines identified and dated elsewhere^15,41–44^ were mapped. Cross-checks were performed against sites that have been revisited (e.g.^45,46^). Subsequently, adjacent and surrounding valleys were examined within the hillshade of the DEM and in Planet^47^ imagery, mostly PlanetScope 3 m resolution https://www.planet.com/explorer/, and within orthorectified 1 m resolution aerial photographs^48^ for contiguous landform evidence.

**Figure 2:**
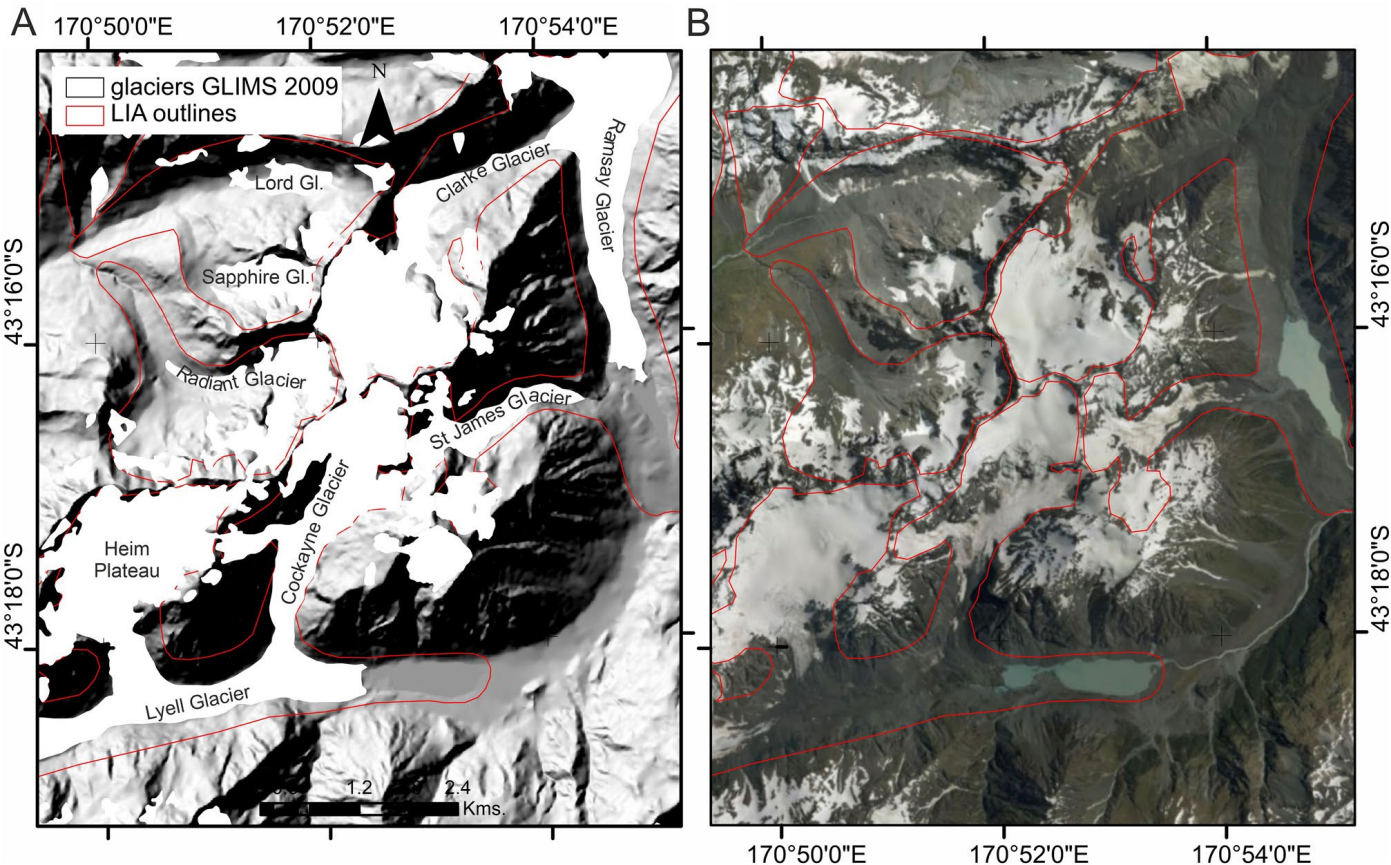
Example from upper Rakaia region of LIA glacier outlines derived from identification of moraines and trimlines as visible on a 8 m resolution digital elevation model (**A**) and high resolution satellite imagery (**B**).

In addition, we computed a (linear) trend surface of the Equilibrium Line Altitude (ELA) for the LIA based on 22 cirque glaciers previously studied^24,49^, which compared favourably with the neoglacial ELA proposed by Porter^50^ for a transect between Mt Cook and Jollie Glacier (Fig. SI 1). By discriminating the land above this LIA ELA trend surface, we scrutinised the landscape to find many low-relief moraines, trimlines (Fig. 2), and more subtle geomorphological evidence that helped us to infer a LIA glacier outline for a total of 400 glaciers. The subtle evidence included stripped bedrock at elevations beneath surrounding vegetated hillslopes, locally-depressed treelines, boulder trains running sub-parallel to contours, truncated fan toe slopes and gully heads. Whilst there are > 3,150 glaciers in the Southern Alps in the 1978 inventory, a vast majority constitute smaller portions of formerly larger (and more contiguous) LIA glaciers. We did not map where moraines or trimlines were absent, such as is common in Fiordland for example where the hard crystalline rock and steep slopes makes the creation and preservation of such features unlikely. More details on the volume loss calculations, which are limited to glacier ablation areas (Fig. SI 2), are given in the SI.

For mean volume change rates, a temporal datum during the LIA interval needed to be applied. It has been noted that the Southern Alps glaciers reached their greatest LIA extents at differing times (^24,49,51^, Table SI 1). Therefore, in order to produce a single mean rate of volume change for the whole Southern Alps, we computed three ice volume change rates; a maximum rate using 1800 CE, a middle rate where 1600 CE was applied, and a minimum rate where 1450 CE was chosen. This approach considers the implicit assumptions related to mapping glacier advance activity using landforms that contain uncertainties related to complex depositional histories and dating uncertainties. It is highly unlikely that all glaciers were most advanced at either the earlier or the later date because of the climatic gradients and variety of topographic settings of glaciers across the Southern Alps, but this approach (to take the middle date) is congruent with palaeoclimate evidence of a colder climate during the mid-to-late 2nd millennium CE^52^ and provides the uncertainty bounds of our mean rate.

Errors in area and volume change estimates will result from DEM resolution, LIA identification and digitising. Our area change estimates are subject to uncertainty depending on the DEM (8 m) and optical image (< 1 m) resolution used for digitising LIA glacier outlines, and subjective choices of the most prominent inner moraine and of trimlines. Nevertheless, in the vast majority of cases the geomorphological evidence is distinct, whilst digitising errors for smaller glaciers will have the largest relative effect in area measurement accuracy. For a typical valley glacier in the Southern Alps of 2 km^2^, digitizing errors of one pixel would typically produce an area of ~  ± 2% (depending on glacier shape). Volume uncertainty is far higher; about 20%, given the 3D surface interpolation as explained in the very similar study of glacier volume change since the LIA in NE Greenland^53^, and surface differencing methods, but is not dependent on the choice of DEM (Table SI 2).

We also make a novel attempt to estimate the total volume of ice across the Southern Alps during the LIA by comparing our LIA surface elevations with those of an ice-free DEM (Fig. SI 3). Our ice-free DEM was derived by subtracting contemporary ice thickness from the DEM. Ice thickness across the Southern Alps (see section 2.5 of^54^) was estimated using a perfect-plasticity centre-line model^55^ using the 1978 glacier outlines. That model was tested against dense bed topography data from five mountain glaciers around the world, including the few direct ice thickness measurements of any Southern Alps glaciers at Tasman Glacier (their Figs. 6d and 9o). That model is well-suited to making rapid region-wide estimates of distributed ice thickness for mountain glaciers such as across southern South America^56^ and for the entire Antarctic Peninsula^57^. Year 2019 ice volume was estimated as the difference between our 2019 ice surface, which was derived in the same manner as for the LIA by interpolating between our 2019 outlines, and the ice-free DEM. More methodological detail is given in our Supplementary Information. A summary and depiction of our surface differencing is given in Fig. SI 4.

## Results

Overall, we mapped LIA outlines of 400 Southern Alps glaciers. The vast majority of these represent the coalescence of many, now smaller, glaciers that have fragmented greatly since the nineteenth century. The total area we mapped as ice covered during the LIA was 1,492 km^2^ ± 104 km^2^. If we assume that the 440 km^2^ that we were unable to map has not changed since the LIA because it is mostly comprised of ice above the regional ELA, then combining that with the area that we have mapped produces a total glaciated LIA area of at least 1932 km^2^ ± 135 km^2^. Compared to the 1978 GLIMS inventory area of 1,463 km^2^ we find a loss of at least 24% in glacier ice areal extent since the LIA. For comparison, Chinn^24^ reported that New Zealand’s Southern Alps glaciers (based on 127 glaciers) had lost 38% of their length and 25% of their area since the LIA. Using newly generated glacier outlines for the year 2019 (Fig. SI 5), we computed an ice coverage of 1,021 km^2^, which is 53.8% of the LIA area and 69.8% of the 1978 area. Only 121 km^2^ (12%) of glacier area in 2019 lies within the LIA ablation areas.

There has been major terminal recession of ice between 2009 to 2019; especially where ice is flowing and calving into proglacial lakes. The difference in terminus outlines between 2009 and 2019 far exceeds the 1978 to 2009 changes (Fig. SI 5). One significant change in glacier outlines shows the Mueller Glacier ablation area (Mt Cook region) has split so that the terminus in Mueller Lake is fed solely by Frind Glacier and Huddleston Glacier. A similar situation can be seen at Clarke Glacier in the Upper Rakaia region, which now has a terminus 1.2 km up-valley from its former connection point to Ramsay Glacier (Fig. 2). Many other glaciers across the Southern Alps also now have remnant ice bodies (former ablation tongues) that are entirely disconnected from ice falls and accumulation areas located upslope. Most nunataks have also enlarged as glacier thinning has proceeded in accumulation areas.

**Figure 3:**
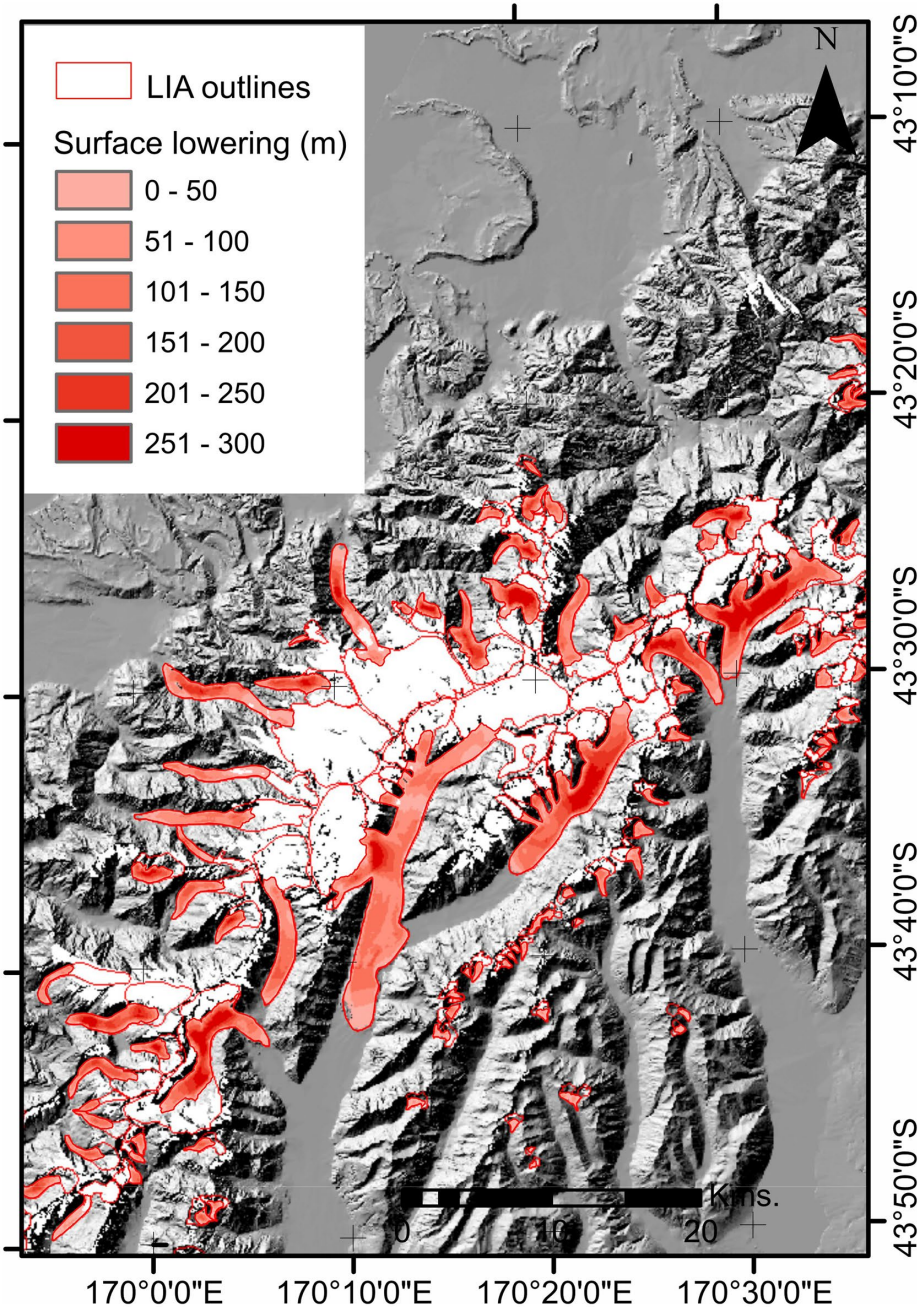
Example from Mt Cook region of surface lowering computed between a LIA ice surface and the contemporary DEM. Note timing of LIA was spatially-variable and chronologically uncertain across the Southern Alps.

Based on the method used herein to calculate volume change of glaciers from a reconstructed LIA ice surface (see SI,^52^), there has been major ice surface elevation lowering (Fig. 3) and consequently at least 60 km^3^ ± 12 km^3^ ice volume loss across the Southern Alps since the LIA (irrespective of which DEM we use; see SI). This lost volume equates to a 41% to 62% of the total LIA volume, which was between 97 km^3^ and 145 km^3^. For comparison, Hoelzle et al.^11^ estimated a slightly larger volume of 170 km^3^ for the Southern Alps during the LIA.

The 2019 volume is estimated in this study to be between 33.7 km^3^ and 50.5 km^3^. We therefore suggest that between the LIA and 2019, 47 to 77% of ice has ablated, and that this estimate is conservative because it takes the model errors into account. We also suggest that between 1978 and 2019, 22% of the 1978 ice volume (which we estimate to be between 43.2 km^3^ and 64. 8 km^3^) was lost, or an additional 14% to 17% of the former LIA volume.

The largest elevation changes (Fig. SI 6) and hence volume loss for any single glacier has been for Tasman Glacier at 4.47 km^3^ ± 0.9 km^3^. Godley has lost 3.49 km^3^ ± 0.7 km^3^, Murchison Glacier has lost 3.47 km^3^ ± 0.7km^3^, and Mueller has lost 2.27 km^3^ ± 0.4 km^3^. These four glaciers (one hundredth of the LIA total number mapped) together account for over one fifth of the total ice mass loss across the Southern Alps since the LIA.

**Figure 4:**
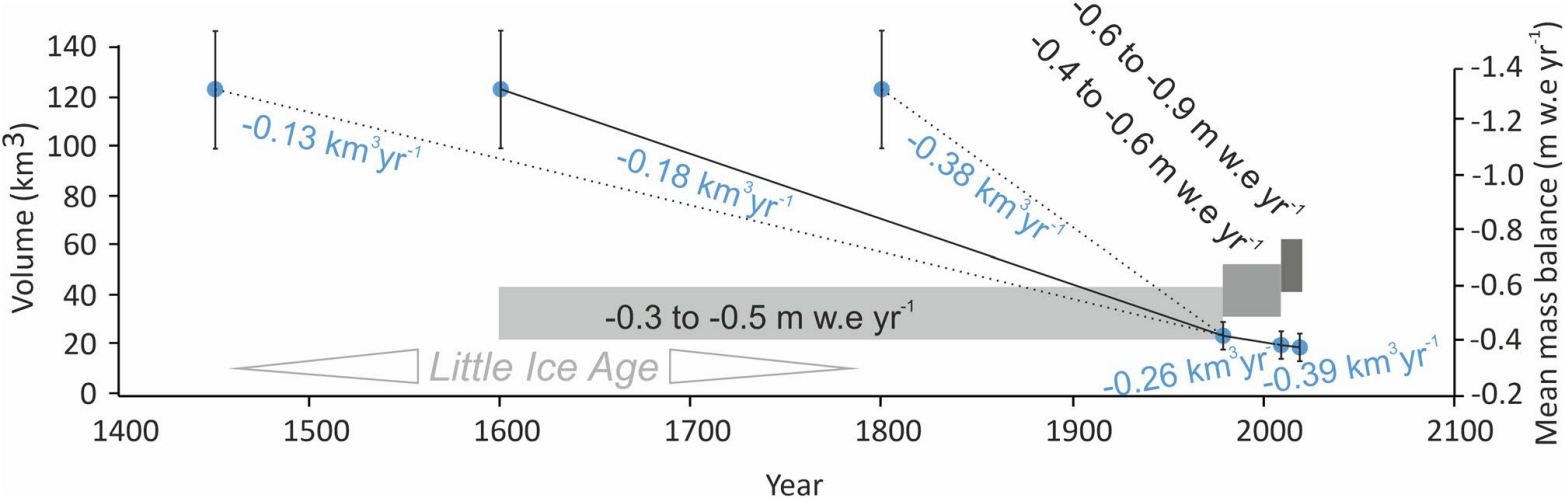
Volume and centennial rate of ice volume change for LIA scenarios considered in this study, compared with modern volume and rates of change for the Southern Alps, New Zealand.

A rate of ice volume loss of 0.13 km^3^ year^−1^ ± 0.1 km^3^ year^−1^ occurred if 1450 CE is taken as the LIA starting point, and from 1600 CE the rate is 0.18 km^3^ year^−1^ ± 0.1 km^3^ year^−1^. The near-end point of the LIA at 1800 CE yields a rate of volume loss of 0.38 km^3^ year^−1^ ± 0.2 km^3^ year^−1^ (Fig. 4). Whilst the start and ends of the LIA interval are marginally subjective when used to calculate these loss estimates, we contend that the middle scenario is conservative and the most plausible, with a given a total mass loss of 109 Gt ± 22 Gt that yields a mean rate of 0.26 Gt year^−1^ ± 0.06 Gt year^−1^. That total mass loss equates to a 0.3 mm sea level rise contribution by Southern Alps glaciers since the LIA at a mean rate of 0.0007 mm year^−1^. We determine a mean annual mass balance of between − 0.3 and − 0.5 m w.e. year^−1^ between the year 1600 and 1978 (Fig. 4). This is rather smaller than the − 1.2 m w.e year^−1^ reduction suggested by Hoelzle et al.^11^ for the LIA to the 1970s but they had a larger LIA volume, a smaller LIA glaciated area and by taking 1850 as the LIA maximum they also considered a much shorter time period.

Ice volume modelled from the 1978 glacier outlines suggests that 44 km^3^ or 81% was contained within 308 glaciers that had an area > 1 km^2^ (Fig. SI 7). The volume estimated from the 2019 outlines is 42.1 km^3^ ± 8.4 km^3^. This implies a rate of volume loss of 0.39 km^3^ year^−1^ ± 0.1 km^3^ year^−1^ over the last decade, equivalent to a mean annual mass balance of − 0.6 to − 0.9 m w.e. year^−1^ (Fig. 4).

## Discussion

There are no global syntheses that estimate cumulative ice volume or mass loss since the time when many mountain glaciers reached sub-maximal positions at the conclusion of the pre-industrial era in the nineteenth century, or the end of the LIA. This study has shown refining the geometry of ice boundaries using moraines and observational data can help to reduce uncertainty in the historic and pre-observation frozen water budget, and also to determine what scale of ice volume loss actually occurred and what ice volume now remains. Our determination of 41 to 62% of the LIA ice volume remaining across the Southern Alps (i.e. 38 to 59% loss) is in good agreement but slightly lower than the estimate of 61% loss by Hoelzle et al.^11^.

In the Southern Hemisphere, Glasser et al.^8^ reported 606 km^3^ lost from the North Patagonian ice field (NPI) and South Patagonian ice field (SPI) collectively, since the LIA. Taking the mid-point of the local LIA timespan in New Zealand of 1,600 CE, our calculated sea level rise contribution from the Southern Alps of 0.0007 mm year^−1^ is 62% of Glasser et al.’s 0.0018 mm year^−1^ for the NPI and 79% of their 0.0034 mm year^−1^ for the SPI. This slower centennial rate of mass loss in the Southern Alps compared to that of Patagonia is most easily explained by the far larger volume of ice in Patagonia than in the Southern Alps.

However, in terms of absolute volume change, it is instructive to note that a total remaining ice volume in southern South America of 5,561 km^3^ ± 1,191 km^3^, for the NPI and SPI combined^56^. Comparing this to the volume change of Glasser et al.^8^ suggests that only 11% of the LIA ice volume of Patagonia has been lost to date. In stark contrast, we find that across the Southern Alps the ice volume lost since the LIA equates to between 41 and 62% of the LIA total. This disproportionate ice loss could be most easily explained by the more northerly location of the Southern Alps in comparison to Patagonia, but probably is also a reflection that the smaller glaciers in New Zealand have faster response times.

Previous work synthesised multi-proxy data to infer regional atmospheric circulation patterns as a driver for pre-industrial New Zealand glacial advances^49,52^. A mean summer atmospheric circulation pattern for 1450 to 1800 CE was derived from quantitative temperature estimates based on the ELAs of 22 glaciers being lower than present, implicating increased frequency of trough synoptic types during the LIA at the expense of blocking types^19^. This glacier-based finding supports prior work that suggested a similar pattern existed for the LIA mean climate state, based on qualitative speleothem temperature and palaeo-water balance interpretations paired with quantitative tree ring temperature reconstructions^52^. Regional ‘trough’ circulation patterns during the LIA were nested within larger hemispheric-scale geopotential and Sea Surface Temperature (SST) anomalies (see Figs. 7 and 8 in ref.^49^) arising from interactions of multiple modes of variability, e.g. El Nino-Southern Oscillation (ENSO), Southern Annular Mode (SAM), Pacific South American (PSA) mode and Zonal Wave 3, which produced cooler regional LIA summer temperatures. More broadly, summer hemispheric-scale spatial field patterns^48^ hint at an important ENSO/SAM contribution, where El Nino Modoki combined with negative SAM, Zonal Wave (ZW) 3–4^58^ and the PSA^59^ to promote regional-scale atmospheric flow that reduced surface air temperatures. The latter two modes of variability, along with the ENSO/SAM dynamic, are recognised as producing heterogeneous surface climate patterns for New Zealand and Patagonia simultaneously, and hence, could have contributed to very different timings and expressions for surface climate and LIA glacier activity at centennial timescales in those regions.

Chinn et al.^32^ estimated that Southern Alps ice volume decreased from 54.5 km^3^ in 1976 to 46.1 km^3^ by 2008. Our volume estimate for the year 1978 is in excellent agreement: we also calculate 54 km^3^ (43.2–64.8 km^3^). From this amount, a mean rate of 0.3 km^3^ year^−1^ volume loss from the mid-1970s to the mid-2000s is double that of our mid-scenario centennial rate (0.18 km^3^ year^−1^) of ice volume loss since the LIA (Fig. 4).

Recent work^25^ investigated the nature of purported New Zealand glacier advances during the 1980s and 1990s in more detail, which constituted a significant proportion of glaciers that were seemingly bucking the global ice retreat trend of the late twentieth Century. That investigation showed the predominance of regionally cooler SSTs in the Tasman Sea, increased prevalence of southerly flow from increased frequency of trough synoptic types, and cooler air temperatures countered impacts of a long-term national warming trend^59^. While the regional cooling allowed New Zealand glaciers to apparently advance during a period of global warming, precipitation changes were determined to be less important during this time. The sensitivity of South Island regional climate conditions to the influence of both tropical (ENSO) and extra-tropical circulation, including SAM and hemispheric-scale PSA and ZW3 wave patterns, was noted. Mackintosh et al.^25^ also concluded that by 2011 the situation had clearly reversed, and significant warm episodes were contributing to the rapid decline of Southern Alps ice volume.

Furthermore, as highlighted (for the decadal time frame^32^) and as we have also found for the centennial time frame, approximately three-quarters of the total Southern Alps ice volume loss has occurred from the (~ 10) largest glaciers, which have a long (protracted) response time. Thus, whilst rapid retreat and glacier length decreases and area loss can be reported for the vast majority of New Zealand’s glaciers, the most salient points in terms of volume and mass and meltwater generation are that (i) committed losses (cf.^60^) for future decades are volumetrically large as a proportion of how much ice remains, and (ii) the overall percentage of ice loss relative to an initial state will likely diminish. Our analysis of ice volume in the Southern Alps remaining since the LIA therefore supports the suggestion^61^ that glaciers in climatically-sensitive regions are close to (or even past) peak glacial meltwater contributions to downstream hydrological systems. Nevertheless, many questions still remain about how meltwater contributions in glacierised catchments will contribute to rivers, and how it can help buffer extreme seasonal climate conditions.

Overall, we suggest the Southern Alps rate of ice volume loss has recently accelerated. The glaciers there are now mostly characterised by; (i) small (< 1 km^2^) rapid-response glaciers progressively becoming restricted to high-elevation land close to the ELA, (ii) topographic shading and topographically-assisted mass inputs via avalanching and wind-blown snow becoming progressively more important proportionally as glacier area becomes restricted beneath steep headwalls, as found for European Alps glaciers^62,63^, and (iii) large valley floor glacier ablation tongues are stagnating beneath increasingly extensive, thick debris covers, but are continuing to lose mass fast because they are at low elevation and are influenced by proglacial lake interactions—including negative feedbacks associated with water contact ablation and tabular calving in front of the local grounding line.

## Conclusions

This study has produced a glacier inventory for NZ composed of glacier outlines pertaining to the nineteenth Century maximum ice extent, coeval with the end of the northern European LIA, with calculations of glacierised area and ice volume loss estimates. It has also created a distributed ice thickness, volume and bed (ice-free) topography across Southern Alps using the 1978 glacier outlines, and year 2019 glacier outlines, area and volume estimates. These spatially-distributed data complement a recent reconstruction of the last glacial maximum ice extent across the Southern Alps using similar methods^64^.

We have determined an acceleration in the rate of volume loss and a progressively more negative mean annual balance from the LIA (centennial scale) to recent decades (Fig. 4). This acceleration is somewhat unsurprising given records of accelerating ice mass loss from other parts of the world under rising temperatures. We realise that the Southern Alps has a much larger proportion of volume loss but a slower absolute rate of loss than in Patagonia (no matter which date of LIA is used for the Southern Alps). This heterogeneity between regions of the Southern Hemisphere is expected on centennial and finer timescales, and it highlights the need for more detailed (i) regionally-differentiated chronologies and (ii) earth surface process studies (e.g. landscape, hydrology, mass balance, dating) that in combination are vitally important for contextualising present conditions and predicting future responses in a warming world.

Across the Southern Alps, former LIA ablation areas are now almost completely ice-free but the exceptions are the very large near-stagnant debris-covered ablation tongues that sit on valley floors at relatively low elevation. These large ablation tongues often terminate in proglacial lakes, which can exacerbate ice loss. The remaining ice at high-elevation is increasingly fragmented, thinning and disconnected from ablation areas thereby exposing increasingly large nunataks. These local glaciological controls determine the exceptionally dynamic proglacial geomorphology^65^, sedimentology and net sandur or outwash plain elevation changes (e.g.^66^). In particular, proglacial lake development affects glacier evolution, interrupts the flux of meltwater and sediments down valley^67,68^ and can control longer-term landscape evolution^69^. Our findings suggest that the timing of peak glacial meltwater production will soon be passed across the Southern Alps, if it has not been already, but we note that the associated spatio-temporal yields of proglacial sediment, minerals and nutrients have yet to be examined.

## Supplementary information

Supplementary information 1.

Dataset S1.

Dataset S2.

## References

[1] Raper S, Braithwaite R (2006). Low sea level rise projections from mountain glaciers and icecaps under global warming. Nature.

[2] Gardner AS, Moholdt G, Cogley JG, Wouters B, Arendt AA, Wahr J, Berthier E, Hock R, Pfeffer WT, Kaser G, Ligtenberg SR (2013). A reconciled estimate of glacier contributions to sea level rise: 2003 to 2009. Science.

[3] Zemp M, Huss M, Thibert E, Eckert N, McNabb R, Huber J, Barandun M, Machguth H, Nussbaumer SU, Gärtner-Roer I, Thomson L (2019). Global glacier mass changes and their contributions to sea-level rise from 1961 to 2016. Nature.

[4] Chen JL, Wilson CR, Tapley BD (2013). Contribution of ice sheet and mountain glacier melt to recent sea level rise. Nat. Geosci..

[5] Zemp M, Frey H, Gärtner-Roer I, Nussbaumer SU, Hoelzle M, Paul F, Haeberli W, Denzinger F, Ahlstrøm AP, Anderson B, Bajracharya S (2015). Historically unprecedented global glacier decline in the early 21st century. J. Glaciol..

[6] Huss M, Hock R (2015). A new model for global glacier change and sea-level rise. Front. Earth Sci..

[7] Benn DI, Owen LA, Osmaston HA, Seltzer GO, Porter SC, Mark B (2005). Reconstruction of equilibrium-line altitudes for tropical and sub-tropical glaciers. Quatern. Int..

[8] Glasser NF, Harrison S, Jansson KN, Anderson K, Cowley A (2011). Global sea-level contribution from the Patagonian Icefields since the Little Ice Age maximum. Nat. Geosci..

[9] Carrivick JL, Davies BJ, Glasser NF, Nývlt D, Hambrey MJ (2012). Late-Holocene changes in character and behaviour of land-terminating glaciers on James Ross Island, Antarctica. J. Glaciol..

[10] Mark BG, Seltzer GO, Rodbell DT, Goodman AY (2002). Rates of deglaciation during the last glaciation and Holocene in the Cordillera Vilcanota-Quelccaya Ice Cap region, southeastern Peru. Quatern. Res..

[11] Hoelzle M, Chinn TJ, Stumm D, Paul F, Zemp M, Haeberli W (2007). The application of glacier inventory data for estimating past climate change effects on mountain glaciers: a comparison between the European Alps and the Southern Alps of New Zealand. Global Planet. Change.

[12] Turney CS, McGlone MS, Wilmshurst JM (2003). Asynchronous climate change between New Zealand and the North Atlantic during the last deglaciation. Geology.

[13] Lynch-Stieglitz J (2004). Hemispheric asynchrony of abrupt climate change. Science.

[14] Schaefer JM, Denton GH, Kaplan M, Putnam A, Finkel RC, Barrell DJ, Andersen BG, Schwartz R, Mackintosh A, Chinn T, Schlüchter C (2009). High-frequency Holocene glacier fluctuations in New Zealand differ from the northern signature. Science.

[15] Putnam AE, Denton GH, Schaefer JM, Barrell DJ, Andersen BG, Finkel RC, Schwartz R, Doughty AM, Kaplan MR, Schlüchter C (2010). Glacier advance in southern middle-latitudes during the Antarctic Cold Reversal. Nat. Geosci..

[16] Shakun JD, Carlson AE (2010). A global perspective on Last Glacial Maximum to Holocene climate change. Quatern. Sci. Rev..

[17] PAGES2k Consortium (2013). Continental-scale temperature variability over the Common Era. Nat. Geosci..

[18] Hall BL, Lowell TV, Bromley GRM, Denton GH, Putnam AE (2019). Holocene glacier fluctuations on the northern flank of Cordillera Darwin, southernmost South America. Quatern. Sci. Rev..

[19] Kidson JW (2000). An analysis of New Zealand synoptic types and their use in defining weather regimes. Int. J. Climatol..

[20] Cohen L, Dean S, Renwick J (2013). Synoptic weather types for the Ross Sea region, Antarctica. J. Climate.

[21] Jiang N, Griffiths G, Lorrey A (2013). Influence of large-scale climate modes on daily synoptic weather types over New Zealand. Int. J. Climatol..

[22] Fauchereau NC, Pohl B, Lorrey AM (2016). Extra-tropical impacts of the Madden-Julian Oscillation over New Zealand from an atmospheric circulation regime perspective. J. Clim..

[23] Lorrey AM, Fauchereau NC (2018). Southwest Pacific atmospheric weather regimes: linkages to ENSO and extra-tropical teleconnections. Int. J. Climatol..

[24] Chinn TJ (1996). New Zealand glacier responses to climate change of the past century. NZ J. Geol. Geophys..

[25] Mackintosh AN, Anderson BM, Lorrey AM, Renwick JA, Frei P, Dean SM (2017). Regional cooling caused recent New Zealand glacier advances in a period of global warming. Nat. Commun..

[26] Barrell, D.J.A. Quaternary glaciers of New Zealand. In *Developments in Quaternary Sciences*, 15, 1047–1064) (Elsevier, 2011)

[27] Putnam AE, Schaefer JM, Denton GH (2012). Regional climate control of glaciers in New Zealand and Europe during the pre-industrial Holocene. Nat. Geosci..

[28] Chinn TJ (1999). New Zealand glacier response to climate change of the past 2 decades. Global Planet. Change.

[29] Carrivick JL, Chase SE (2011). Spatial and temporal variability of annual glacier equilibrium line altitudes in the Southern Alps, New Zealand. NZ J. Geol. Geophys..

[30] Hochstein MP, Claridge D, Henrys SA, Pyne A, Nobes DC, Leary SF (1995). Downwasting of the Tasman Glacier, South Island, New Zealand: changes in the terminus region between 1971 and 1993. NZ J. Geol. Geophys..

[31] Quincey DJ, Glasser NF (2009). Morphological and ice-dynamical changes on the Tasman Glacier, New Zealand, 1990–2007. Global Planet. Change.

[32] Chinn T, Fitzharris BB, Willsman A, Salinger MJ (2012). Annual ice volume changes 1976–2008 for the New Zealand Southern Alps. Global Planet. Change.

[33] Chinn TJH (2001). Distribution of the glacial water resources of New Zealand. J. Hydrol. (NZ).

[34] GLIMS (2019). Randolph Glacier Inventory https://www.glims.org/RGI/index.html last visited December, 2019.

[35] Sirguey, P. & More, B. GLIMS Glacier Database. Boulder, NSIDC (2010)

[36] Land Information New Zealand (LINZ). NZ 8m Digital Elevation Model (2012), https://data.linz.govt.nz/layer/51768-nz-8m-digital-elevation-model-2012/ last visited September 2019 (2019a).

[37] Wardle P (1973). Variations of the glaciers of Westland National Park and the Hooker Range, New Zealand. NZ J. Bot..

[38] Winkler S (2000). The ‘Little Ice Age’ maximum in the Southern Alps, New Zealand: preliminary results at Mueller Glacier. Holocene.

[39] Winkler S (2004). Lichenometric dating of the ‘Little Ice Age’ maximum in Mt Cook National Park, Southern Alps, New Zealand. Holocene.

[40] Reznichenko NV, Davies TR, Winkler S (2016). Revised palaeoclimatic significance of Mueller Glacier moraines, Southern Alps, New Zealand. Earth Surf. Proc. Land..

[41] Burrows CJ (1973). Studies on some glacial moraines in New Zealand—2: ages of moraines of the Mueller, Hooker and Tasman glaciers (S79). NZ J. Geol. Geophys..

[42] Burrows CJ (1975). Late Pleistocene and Holocene moraines of the Cameron Valley, Arrowsmith Range, Canterbury, New Zealand. Arctic Alpine Res..

[43] Bacon SN, Chinn TJ, Van Dissen RJ, Tillinghast SF, Goldstein HL, Burke RM (2001). Paleo-equilibrium line altitude estimates from late Quaternary glacial features in the Inland Kaikoura Range, South Island, New Zealand. NZ J. Geol. Geophys..

[44] Dowling, L.H. The holocene glacial history of dart glacier, Southern Alps, New Zealand. MSc thesis at Victoria University, Wellington, NZ, 135 (2019)

[45] McKinzey KM, Lawson W, Kelly D, Hubbard A (2004). A revised Little Ice Age chronology of the Franz Josef Glacier, Westland, New Zealand. J. R. Soc. New Zealand.

[46] Purdie H, Anderson B, Chinn T, Owens I, Mackintosh A, Lawson W (2014). Franz Josef and Fox Glaciers, New Zealand: historic length records. Global Planet. Change.

[47] Planet. Planet Application Program Interface. In Space for Life on Earth. San Francisco, CA. Retrieved from https://api.planet.com (2017)

[48] Land Information New Zealand (LINZ). NZ Aerial Imagery https://data.linz.govt.nz/set/4702-nz-aerial-imagery/ last visited September 2019 (2019b).

[49] Lorrey A, Fauchereau N, Stanton C, Chappell P, Phipps S, Mackintosh A, Renwick J, Goodwin I, Fowler A (2014). The Little Ice Age climate of New Zealand reconstructed from Southern Alps cirque glaciers: a synoptic type approach. Clim. Dyn..

[50] Porter SC (1975). Equilibrium-line altitudes of late quaternary glaciers in the Southern Alps, New Zealand. Q. Res..

[51] Gellatly AF, Chinn TJ, Röthlisberger F (1988). Holocene glacier variations in New Zealand: a review. Quatern. Sci. Rev..

[52] Lorrey A, Williams P, Salinger J (2008). Speleothem stable isotope records interpreted within a multi-proxy framework and implications for New Zealand palaeoclimate reconstruction. Quatern. Int..

[53] Carrivick JL, Boston CM, King O, James WH, Quincey DJ, Smith MW, Grimes M, Evans J (2019). Accelerated volume loss in glacier ablation zones of NE Greenland, Little Ice Age to present. Geophys. Res. Lett..

[54] James, W.H.M. A landform based 3D reconstruction of glacier ice at the Last Glacial Maximum in the Southern Alps, New Zealand (Doctoral dissertation, University of Leeds) (2016).

[55] James WH, Carrivick JL (2016). Automated modelling of spatially-distributed glacier ice thickness and volume. Comput. Geosci..

[56] Carrivick JL, Davies BJ, James WH, Quincey DJ, Glasser NF (2016). Distributed ice thickness and glacier volume in southern South America. Global Planet. Change.

[57] Carrivick JL, Davies BJ, James WH, McMillan M, Glasser NF (2019). A comparison of modelled ice thickness and volume across the entire Antarctic Peninsula region. Geografiska Ann. Ser. A Phys. Geography.

[58] Raphael MN (2004). A zonal wave 3 index for the Southern Hemisphere. Geophys. Res. Lett..

[59] Mo KC, Paegle JN (2001). The Pacific-South American modes and their downstream effects. Int. J. Climatol..

[60] Mernild SH, Lipscomb WH, Bahr DB, Radić V, Zemp M (2013). Global glacier changes: a revised assessment of committed mass losses and sampling uncertainties. The Cryosphere.

[61] Huss M, Hock R (2018). Global-scale hydrological response to future glacier mass loss. Nat. Clim. Change.

[62] Carrivick JL, Berry K, Geilhausen M, James WH, Williams C, Brown LE, Rippin DM, Carver SJ (2015). Decadal-scale changes of the Ödenwinkelkees, central Austria, suggest increasing control of topography and evolution towards steady state. Geografiska Ann. Ser. A Phys. Geography.

[63] Huss M, Fischer M (2016). Sensitivity of very small glaciers in the Swiss Alps to future climate change. Front. Earth Sci..

[64] James WH, Carrivick JL, Quincey DJ, Glasser NF (2019). A geomorphology based reconstruction of ice volume distribution at the last glacial maximum across the Southern Alps of New Zealand. Quatern. Sci. Rev..

[65] Carrivick JL, Heckmann T (2017). Short-term geomorphological evolution of proglacial systems. Geomorphology.

[66] Carrivick JL, Rushmer EL (2009). Inter-and intra-catchment variations in proglacial geomorphology: an example from Franz Josef Glacier and Fox Glacier, New Zealand. Arct. Antarct. Alp. Res..

[67] Carrivick JL, Tweed FS (2013). Proglacial lakes: character, behaviour and geological importance. Quatern. Sci. Rev..

[68] Carrivick JL, Rushmer EL (2006). Understanding high-magnitude outburst floods. Geol. Today.

[69] Sutherland JL, Carrivick JL, Shulmeister J, Quincey DJ, James WH (2019). Ice-contact proglacial lakes associated with the last glacial maximum across the Southern Alps, New Zealand. Quatern. Sci. Rev..

