# Supplementary material for: Ice thickness and volume changes across the Southern Alps, New Zealand, from the little ice age to present
Source: Sci Rep. 2020 Aug 7;10:13392. doi: 10.1038/s41598-020-70276-8 (PMC7415160; doi:10.1038/s41598-020-70276-8)
Supplement: Supplementary file 1 — Supplementary information 1. [file 41598_2020_70276_MOESM1_ESM.docx]

Supporting Information for

**Ice thickness and volume changes across the Southern Alps, New Zealand, from the Little Ice Age to present**

**Jonathan L. Carrivick^1^, William H.M., James^1^, Michael Grimes^1^,**

**Jenna L. Sutherland^1^, Andrew M. Lorrey^2^**

^1^School of Geography, University of Leeds, Woodhouse Lane, Leeds, West Yorkshire, LS2 9JT, UK.

^2^National Institute of Water and Atmospheric Research LTD, Auckland, New Zealand.

**Contents of this file**

- Text on LIA glacier geometry reconstruction methods
- Text on year 2019 glacier outlines: derivation from Sentinel imagery
- Figures SI 1 to SI 7
- Table SI 1 and SI 3

**Additional Supporting Information (Files uploaded separately)**

- **Data Set S1.** Our LIA glacier outlines and ablation areas, and our historical glacier outlines (1978, 2009, 2019) are made freely available as ArcGIS shapefiles in NZGD2000 NZTM projection.
- **Data Set S2.** Our LIA glacier ablation area surface elevation and lowering maps and our contemporary ice thickness maps are made freely available as ArcGIS raster grids in NZGD2000 NZTM projection.

**LIA dates of glacier moraines in the Southern Alps**

| **Age** | **Method** | **Reference** |
| --- | --- | --- |
| Main maxima appear to have occurred before about 1620CE, 1780CE, and 1830CE | Counts of growth rings of trees and shrubs, lichenometry, stages of development of vegetation, and ^14^C determinations. | [37] Wardle, P., 1973. Variations of the glaciers of Westland National Park and the Hooker Range, New Zealand. *New Zealand Journal of Botany*, *11*(2), pp.349-388. |
| Marked expansions of glaciers in mid 15th century CE, mid 16th century CE, early and mid-to-late 17th century CE, mid and late 18th century CE, early, mid, and late 19th century CE, plus one early 20^th^ century CE | Lichen measurements, geomorphology, vegetation cover and degree of weathering of rocks | [41] Burrows, C.J., 1973. Studies on some glacial moraines in New Zealand – 2: ages of moraines of the Mueller, Hooker and Tasman glaciers (S79). *New Zealand Journal of Geology and Geophysics*, 16(4), pp.831-856. |
| Set of eight ‘Arrowsmith moraines’ dated to between 1420CE and 1890CE, plus one at 1930CE. | Radiocarbon dates and inferred correlation with dated sequences in adjacent areas | [42] Burrows, C.J., 1975. Late Pleistocene and Holocene moraines of the Cameron Valley, Arrowsmith Range, Canterbury, New Zealand. *Arctic and Alpine Research*, 7(2), pp.125-140. |
| Little Ice Age maximum about ad 1725/1730CE;  Major readvances during the second half of the nineteenth century (around AD 1860 and 1890/95) | Lichenometric-dating and comparison with Schmidt hammer measurements | [38] Winkler, S., 2000. The ‘Little Ice Age’ maximum in the Southern Alps, New Zealand: preliminary results at Mueller Glacier. *The Holocene*, *10*(5), pp.643-647.  [39] Winkler, S., 2004. Lichenometric dating of the ‘Little Ice Age’ maximum in Mt Cook National Park, Southern Alps, New Zealand. *The Holocene*, *14*(6), pp.911-920. |
| 400-300 years ago | Geomorphological mapping and cosmogenic isotope dating | Eaves, S., pers. comm.  [44] Dowling, L., 2019. The Holocene Glacial History of Dart Glacier, Southern Alps, New Zealand. Victoria University of Wellington thesis. |
| 1800-1700, 1500 | Radiocarbon dating of buried wood and soils | [51] Gellatly, A.F., Chinn, T.J. and Röthlisberger, F., 1988. Holocene glacier variations in New Zealand: a review. *Quaternary Science Reviews*, *7*(2), pp.227-242. |
| Large portions of inner most Mueller Glacier terminal moraine suite constrained between ~1400-1775CE with positions a few meters to a few hundred meters inboard of mid- and late-Holocene landforms | Cosmogenic isotope dating | [14] Schaefer, J.M., Denton, G.H., Kaplan, M., Putnam, A., Finkel, R.C., Barrell, D.J., Andersen, B.G., Schwartz, R., Mackintosh, A., Chinn, T. and Schlüchter, C., 2009. High-frequency Holocene glacier fluctuations in New Zealand differ from the northern signature. *Science*, *324*(5927), pp.622-625. |
| Several moraines dated between 1300-1700CE with positions a few meters to a few hundred meters inboard of mid- and late-Holocene landforms. Additional eyewitness accounts show terminal position of glacier meters inboard of this moraine suite and a minimal/absent proglacial lake | Cosmogenic isotope dating | [27] Putnam, A.E., Schaefer, J.M., Denton, G.H., Barrell, D.J., Finkel, R.C., Andersen, B.G., Schwartz, R., Chinn, T.J. and Doughty, A.M., 2012. Regional climate control of glaciers in New Zealand and Europe during the pre-industrial Holocene. *Nature Geoscience*, *5*(9), pp.627-630. |
| Moraine on the southwest slope of Mt Ruapehu dated to ~1800CE, and additional, abutting landforms upslope (younger) | Cosmogenic isotope dating | [70] Eaves, S.R., Winckler, G., Mackintosh, A.N., Schaefer, J.M., Townsend, D.B., Doughty, A.M., Jones, R.S. and Leonard, G.S., 2019. Late-glacial and Holocene glacier fluctuations in North Island, New Zealand. *Quaternary Science Reviews*, 223, p.105914. |

**Table SI 1.** Review of dates of glacier moraines in the Southern Alps assigned to the LIA, see also reviews in Chinn (1996) [24], Lorrey et al. (2008, 2014) [49, 52]. Recent results from cosmogenic studies of Eaves and Winkler are drawn from the ICE-D Alpine database

**LIA glacier geometry reconstruction methods for Southern Alps**

1. In ArcMap (v. 10.4.1) and using spatial analyst extension, we mapped the LIA limits of [37], plus other literature (see table SI 1) with LIA dates.
2. For glaciers > 1 km^2^ (n = 410) we mapped contiguous moraines and trimlines using 8 m DEM (LINZ) and 1 m aerial photograph mosaic (LINZ), using elevations of moraine crests and trimlines (vegetation, sediment veneer, some stream gully head elevations) to assist in estimating glacier outline on opposing valley sides where necessary.
3. We mapped glacier-specific ELAs at the LIA determined by [24] as modified by [49].
4. Made ELA trend surface for LIA from the 22 glaciers of [49], plus those refs. in Table SI 1 and checked versus Porter (1975) [50] ‘neoglacial’ transect between Mt Cook (ELA ~ 1600 m.a.s.l at LIA) and Jollie stream (ELA ~ 1850 m.a.s.l at LIA) (Fig. SI 1).
5. Determined land above ELA trend surface (Fig. SI 2) and checked for any missed geomorphology, now included stripped bedrock and large boulder clusters aligned parallel to contours across valley floors (but without a corresponding ridge visible in the DEM), especially within empty cirques or trough valleys at same elevation as moraines and trimline evidence already identified. But, else erred on conservative approach because GLIMS could have some snow patches included and because very thin hanging glaciers do not tend to produce moraines and are volumetrically unimportant.
6. Most ambiguous parts are in south west where hard geology not very conducive to moraine or (depositional) trimline formation and where forest growth is intense.
7. Due to ambiguity of evidence at some modern glaciers causing them to be excluded, and inclusion of some obvious contiguous evidence in neighbouring valleys, final number of LIA glaciers identified n = 400. Note many of these LIA glaciers are a coalescing of several modern glaciers (Fig. SI 3).
8. ‘Densify’ ablation area outlines to add vertices evenly along all polygon edges (else bit we digitised versus bit from GLIMS will be very different).
9. ‘Simplify polygon’ to remove extraneous LIA polygon vertices [and thus speed up subsequent computations].
10. Converted ‘feature vertices to points’.
11. ‘Extract values to point’ from DEM.
12. ‘Interpolate ablation area points using extracted rastervalue to LIA surface’ [After testing NN, IDW, spline, kriging) we used NN as most realistic. Kriging gave similar results but very computationally intensive]
13. LIA_surface minus DEM = surface lowering.
14. Use zonal statistics to determine sum elevation change for each glacier and multiply by cell size to get volume change per glacier.

We did not map LIA outlines where geomorphological evidence was absent or ambiguous, such as (i) in Southland and Fiordland where a range of factors (including hard crystalline geology, high rainfall and very steep slopes) are unfavourable for moraine creation and preservation, and (ii) for small and steep hanging glaciers (permanent ice at altitude that is not situated in a well-defined cirque or depression). We consider this study has estimated LIA outlines for ~ 75 % of the land area around the LIA ELA.

Although we made a conservative estimate of LIA glaciated area, we can account for the majority ice volume lost since the LIA because it will have been located below the LIA equilibrium line altitude (ELA). In addition, the large valley glaciers (Tasman Glacier, Murchison Glacier, Mueller Glacier, Hooker Glacier, Fox Glacier, Franz Josef Glacier) account for a large proportion of ice volume in the Southern Alps [24]. We calculated the change in glacier volume from the LIA to the present day by differencing a LIA glacier ablation area surface with the modern DEM. Following the method of Carrivick et al. (2019a) [53], LIA glacier ablation areas were produced by automatically estimating glacier-specific equilibrium line altitudes (ELA) using an the Area-Altitude Balance Ratio (AABR) method within the ArcGIS tool developed by Pellitero et al. (2015) [71]. The AABR method was chosen to explicitly consider (spatially-distributed) glacier hypsometry [72]. These glacier-specific ELAs were subsequently merged with our mapped moraine crests and trimlines to create a single polygon for each glacier ablation area. With insufficient data (i.e. mass balance measurements) available to parameterise the AA balance ratios (BRs) directly, we estimated the ELAs using two scenarios based on representative BRs. One scenario was with a BR of 1.75 for all Southern Alps glaciers as suggested to be typical of mountain glaciers by Rea (2009) [72] and as recommended by Pellitero et al. (2015) [71]. The other scenario was with a BR = 1.9 for ‘maritime’ glaciers to the west of the main divide and 1.59 for ‘continental mid-latitude’ glaciers to the east, where those values are derived from the European Alps and mindful of the east-west glaciation limit and climate gradients across the Southern Alps [29, 50, 73]. There was very little difference in the ELAs and hence the ablation areas produced by these two BR scenarios (Fig. SI 2). They both fitted the geomorphological evidence, i.e. the maximum height of lateral moraines, which is a good proxy for the glacier-specific ELA [74]. Surface lowering was converted to a volume change estimate by summing the grid cell elevation changes for each glacier zone and multiplying by cell size.

Volume change estimates presented in this study can only be considered as a proxy for mass balance if it is assumed that glacier surfaces above the ELA have not changed since the LIA. Thus, volume change using the methods in this work should be considered as minimum estimates because probably some mass loss has occurred above (LIA) ELAs as they have risen in elevation due to atmospheric 20^th^ Century warming. That mass loss above the (LIA) ELA would probably be via surface lowering, rather than areal contraction, and so we would not detect it with our mapping. Effects of precipitation on Southern Alps glaciers through the 20^th^ Century, which above the ELA could mean vast amounts of snowfall, are very poorly known. Our workflow also pertains only to glaciers where GLIMS outlines exist. We note there are many empty cirques that may have held small glaciers during the LIA (Fig. SI 2), and this ice contribution is not included in LIA-present volume change estimates.

Errors in area and volume change estimates will result from DEM resolution, LIA identification and digitising. Our LIA area change estimates are subject to uncertainty depending on the hillshaded DEM (8 m) and optical image (< 1 m) resolution used for digitising moraine crests, and subjective choices of the most prominent inner moraine and of trimlines. Nevertheless, in the vast majority of cases the geomorphological evidence is distinct, whilst digitising errors for smaller glaciers will have the largest relative effect in area measurement accuracy [75]. For a typical valley glacier in the Southern Alps of 2 km^2^, digitizing errors of one pixel would typically produce an area of ~ ± 2 % (depending on glacier shape), which is less than Nuimura et al. (2015) [75] reported for their Himalaya glaciers but they were using 30 m raster imagery and included inexperienced operators and poorly-lit imagery. For the debris-covered ablation tongues, uncertainty might be as much as 10 % [75].

Uncertainty in our 2019 outlines is probably similar to that described above for our LIA outlines, but given the 10 m and 20 m resolution Sentinel imagery used to automatically derive these outlines we conservatively estimate a few pixel width error in outlines and hence ± 5 % in glacier area.

Whilst no analysis of DEM uncertainty has been made for New Zealand, a study by Santillan and Santillan (2016) [76] found that the ALOS DEM has the lowest Root Mean Square Error (RMSE) of 5.68 m, followed by SRTM-30m (RMSE = 8.28 m), and ASTER GDEM2 (RMSE = 11.98 m). There are no available uncertainty estimates of the LINZ (2019a) 8 m DEM though it is known that this is composite of different data sources and time periods. We therefore conducted an assessment of the effect of DEM source on our volume estimates. Our LIA volume estimate and our 2019 volume estimate each have bounds of uncertainty that do not depend on DEM source (see screenshot of different DEM model runs below).

| DEM source | grid res. | time frame | assigned year | ablation area at LIA (km^2^) | vol. change to DEM (km^3^) | mean rate of vol. change (km^3^yr^-1^) | Mean annual mass balance (m w.e yr^-1^) if from year 1600 |
| --- | --- | --- | --- | --- | --- | --- | --- |
| LINZ | 8 | 1980s to 2010 | 1995 | 609.95 | -59.3 | -0.030 | -0.06 |
| ALOS | 30 | 2006 to 2011 | 2008 | 603.26 | -62.14 | -0.031 | -0.07 |
| ASTER | 30 | 2000 to 2013 | 2000 | 603.31 | -61.47 | -0.031 | -0.07 |
| SRTM | 30 | Feb. 2000 | 2007 | 605.91 | -59.4 | -0.030 | -0.06 |

**Table SI 2.** Table evidencing analysis of the effect of DEM (source, time, resolution) on volume estimates and mean rates of volume change.

Our volume estimates do depend on surface interpolation method [c.f. 53]; both ice surface and glacier bed (ice-free) surface, where the latter is derived from a perfect-plasticity ice thickness model [55] and applied regionally to hundreds of mountain glaciers in south America [56] and across the Antarctic Peninsula [57]. It has also been applied to determining bed topography for use in Quaternary Glaciation studies, such as for a reconstruction of the Last Glacial maximum ice across the Southern Alps [64]. If we take +/- 20 % following the volume uncertainty reported by James and Carrivick (2016) [55] for the ice thickness model, then as a conservative estimate that produces a LIA volume range from 97 km^3^ to 145 km^3^ and a 2019 volume range of 33.7 to 50.5 km^3^.

LIA volume = vol. change + contemporary ice within LIA ablation areas + contemporary ice within accumulation areas (Fig. SI 4).

LIA ice volume = 97 km^3^ to 145 km^3^

1978 ice volume = 43.2 km^3^ to 64.8 km^3^

2019 ice volume = 33.7 km^3^ to 50.5 km^3^

Ice volume loss from LIA to 1978:

43.2/145 = 0.297 (30 % remaining, or 60 % loss)

64.8/97 = 0.668 (67 % remaining, or 33 % loss)

Ice volume loss from LIA to 2019

33.7/145 = 0.232 (~23 % remaining, or 77 % loss)

50.5/97 = 0.521 (~53 % remaining or 47 % loss)

These are really conservative estimates of volume loss because they take the model errors into account. These estimates suggest that between 1978 and 2019, an additional 14 to 17 % of what was the former LIA volume was lost.

Evaluating the volume ranges for 1978 to 2019, ~ 22 % loss from 1978 to 2019 is reasonable. One solution for 1978 to 2019 loss also indicates ~ 50 % ice loss may have occurred.

For the purposes of evaluating the relative importance of Southern Alps glaciers to global climate we converted our volume changes into mass changes. For this calculation we used a density of ice of 900 kg/m^3^ [77]. That density assumes for simplicity that all the volume lost was ice, even though a small proportion could have been firn. The mass of ice was converted to a sea level equivalent (SLE) using an ocean area of 3.62 x 10^8^ km^2^ [78].

| Time period | Area change  (km^2^) | Volume change (km^3^) | V-max  (km^3^) | V_min  (km^3^) | Mass (Gt) | SLE (mm) | Total m w.e (m) | Mean annual mass balance (m w.e / yr) | Max.  (m w.e / yr) | Min.  (m w.e / yr) |
| --- | --- | --- | --- | --- | --- | --- | --- | --- | --- | --- |
| **1450 to 1978** | 469 | 116 | 145 | 97 | 99 | 0.27 | 210.23 | 0.38 | 0.47 | 0.31 |
| **1600 to 1978** | 469 | 116 | 145 | 97 | 99 | 0.27 | 210.23 | 0.51 | 0.64 | 0.43 |
| **1850 to 1978** | 469 | 116 | 145 | 97 | 99 | 0.27 | 210.23 | 1.31 | 1.64 | 1.10 |
| **1979 to 2009** | 450 | 8 |  |  | 7 | 0.02 | 15.11 | 0.49 |  |  |
| **2009 to 2019** | 442 | 3.9 |  |  | 3 | 0.01 | 7.50 | 0.75 |  |  |

**Table SI 3.** Volume and mass balance estimates for each time period considered in this study.

**Year 2019 glacier outlines: derivation from Sentinel imagery**

Image pre-processing and classification for year 2019 glacier outlines

- Analysis was conducted in Google Earth Engine, a cloud based parallel computing platform. Sentinel-2 level-2A imagery was used due to its higher resolution (10 m resolution visible and NIR bands, 20 m SWIR bands) when compared to Landsat (30 m spectral bands) and superior image quality during the acquisition dates of interest.

- The Sentinel archive was filtered to produce an image collection containing images acquired between 20th December 2018 and 31^st^ March 2019. This collection was then further filtered based on image metadata to select only images with a ‘cloud pixel percentage’ lower than 1%. The resultant image collection was then mosaicked using a median operator so that the median reflectance value overlapping pixels were selected to reduce the inclusion of cloud (high reflectance) and shadow (low reflectance) for overlapping areas.

-Band ratios were calculated from the mosaicked image as follows:

𝑁𝐷𝑆𝐼=(𝐺𝑟𝑒𝑒𝑛−𝑆𝑊𝐼𝑅1)(𝐺𝑟𝑒𝑒𝑛+𝑆𝑊𝐼𝑅1) (1)

𝑁𝐷𝑉𝐼= (𝑁𝐼𝑅−𝑅𝐸𝐷)(𝑁𝐼𝑅+𝑅𝑒𝑑) (2)

𝑁𝐷𝑊𝐼= (𝐺𝑟𝑒𝑒𝑛−𝑁𝐼𝑅)(𝐺𝑟𝑒𝑒𝑛+𝑁𝐼𝑅) (3)

- A grey-level co-occurrence matrix was produced from the mosaicked Sentinel-2 image to indicate spectral surface texture. A principal component analysis was conducted on five of the textural features (angular second moment, contrast, correlation, inverse difference moment, sum of squares: variance) and first principal component (PC) selected to capture the highest variance across variables within one band value [79, 80].

- The 4 spectrally derived bands (NDVI, NDSI, NDWI, GLCM PC) were stacked into one image which was classified using a supervised Random Forest (RF) classifier with n=50. Explanation of RF classifiers is given in [81, 82]. The resultant classification was aggregated to result in a binary image with classes ‘glaciers’ and ‘other’.

- Mis-classifications were identified by comparing the 2019 outlines to those of 1978 and 2009 and then manually corrected where deemed necessary using expert judgement and interpretation of the raw Sentinel images. Mis-classifications were almost entirely due to parts of debris-covered ablation tongues and avalanche debris, the former of which we identified visually via thermokarst features, such as rugged surface topography [75]. As a final check, all our 2019 outlines are spatially contained within the 1978 outlines

**References (not listed in the main manuscript)**

[70] Eaves, S.R., Winckler, G., Mackintosh, A.N., Schaefer, J.M., Townsend, D.B., Doughty, A.M., Jones, R.S. and Leonard, G.S., 2019. Late-glacial and Holocene glacier fluctuations in North Island, New Zealand. *Quaternary Science Reviews*, 223, p.105914.

[71] Pellitero, R., Rea, B.R., Spagnolo, M., Bakke, J., Hughes, P., Ivy-Ochs, S., Lukas, S. and Ribolini, A., 2015. A GIS tool for automatic calculation of glacier equilibrium-line altitudes. *Computers and Geosciences*, 82, pp.55-62.

[72] Rea, B.R., 2009. Defining modern day Area-Altitude Balance Ratios (AABRs) and their use in glacier-climate reconstructions. *Quaternary Science Reviews*, 28(3-4), pp.237-248.

[73] Henderson, R.D. and Thompson, S.M., 1999. Extreme rainfalls in the Southern Alps of New Zealand. *Journal of Hydrology (New Zealand)*, pp.309-330.

[74] Carrivick, J.L. and Brewer, T.R., 2004. Improving local estimations and regional trends of glacier equilibrium line altitudes. *Geografiska Annaler: Series A, Physical Geography*, 86(1), pp.67-79.

[75] Nuimura, T., Sakai, A., Taniguchi, K., Nagai, H., Lamsal, D., Tsutaki, S., Kozawa, A., Hoshina, Y., Takenaka, S., Omiya, S., Tsunematsu, K., Tshering, P., and Fujita, K.: The GAMDAM glacier inventory: a quality-controlled inventory of Asian glaciers, *The Cryosphere*, 9, 849–864.

[76] Santillan, J.R. and Makinano-Santillan, M., 2016. Vertical accuracy assessment of 30-m resolution ALOS, ASTER and SRTM global DEMs over northeastern Mindanao, Philippines. International Archives of the Photogrammetry, *Remote Sensing & Spatial Information Sciences*, 41.

[77] Huss, M., 2013. Density assumptions for converting geodetic glacier volume change to mass change. *The Cryosphere*, 7(3), pp.877-887.

[78] Hock, R., de Woul, M., Radić, V. and Dyurgerov, M., 2009. Mountain glaciers and ice caps around Antarctica make a large sea‐level rise contribution. *Geophysical Research Letters*, 36(7).

[79] Haralick, R.M., Shanmugam, K. and Dinstein, I.H., 1973. Textural features for image classification. *IEEE Transactions on systems, man, and cybernetics*, (6), pp.610-621.

[80] Marceau, D.J., Howarth, P.J., Dubois, J.M.M. and Gratton, D.J., 1990. Evaluation of the grey-level co-occurrence matrix method for land-cover classification using SPOT imagery. *IEEE Transactions on Geoscience and Remote Sensing*, 28(4), pp.513-519.

[81] Gislason, P.O., Benediktsson, J.A. and Sveinsson, J.R., 2006. Random forests for land cover classification. *Pattern Recognition Letters*, 27(4), pp.294-300.

[82] Pal, M., 2005. Random forest classifier for remote sensing classification. International *Journal of Remote Sensing*, 26(1), pp.217-222.


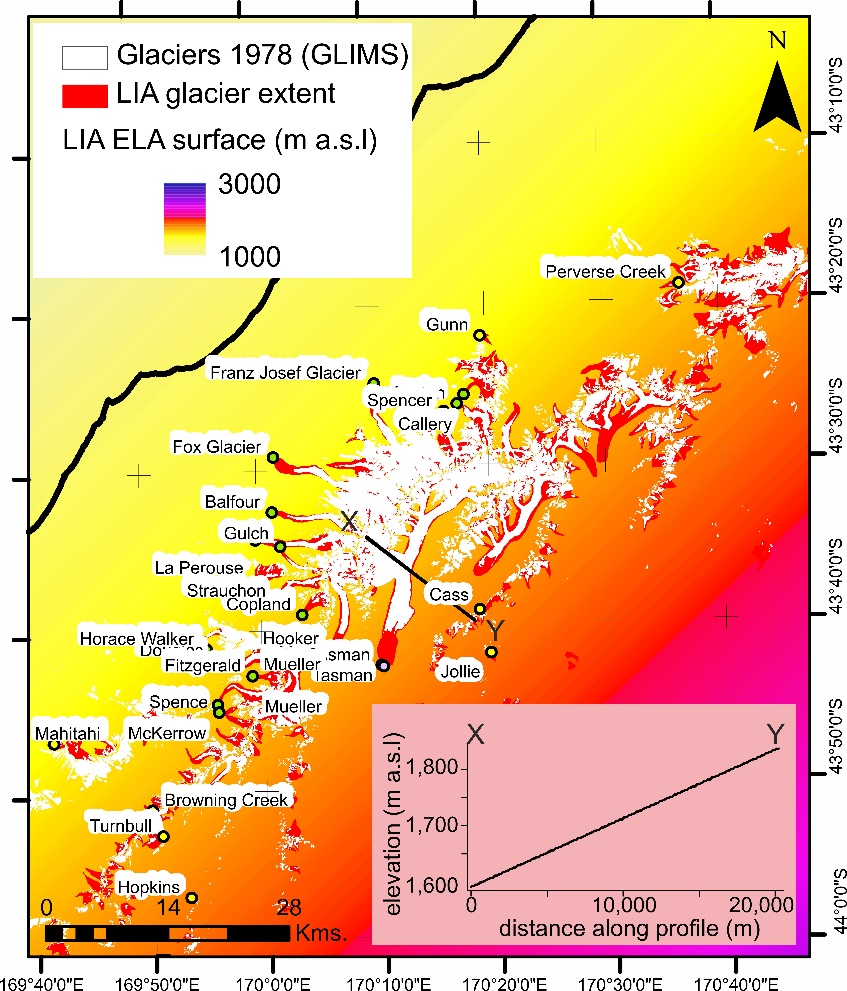


**Figure SI 1.** A linear interpolation between LIA ELAs from 3000 m.a.s.l. to 1000 m.a.s.l. of Lorrey et al. (2014) who modified from Chinn (1996), to produce a regional linear trend surface. Inset graph shows elevation of the ELA trend surface along a transect between Mt Cook and Jollie Glacier, and this profile agrees extremely well (absolute values) with the neoglacial ELA pattern described by Porter (1975).


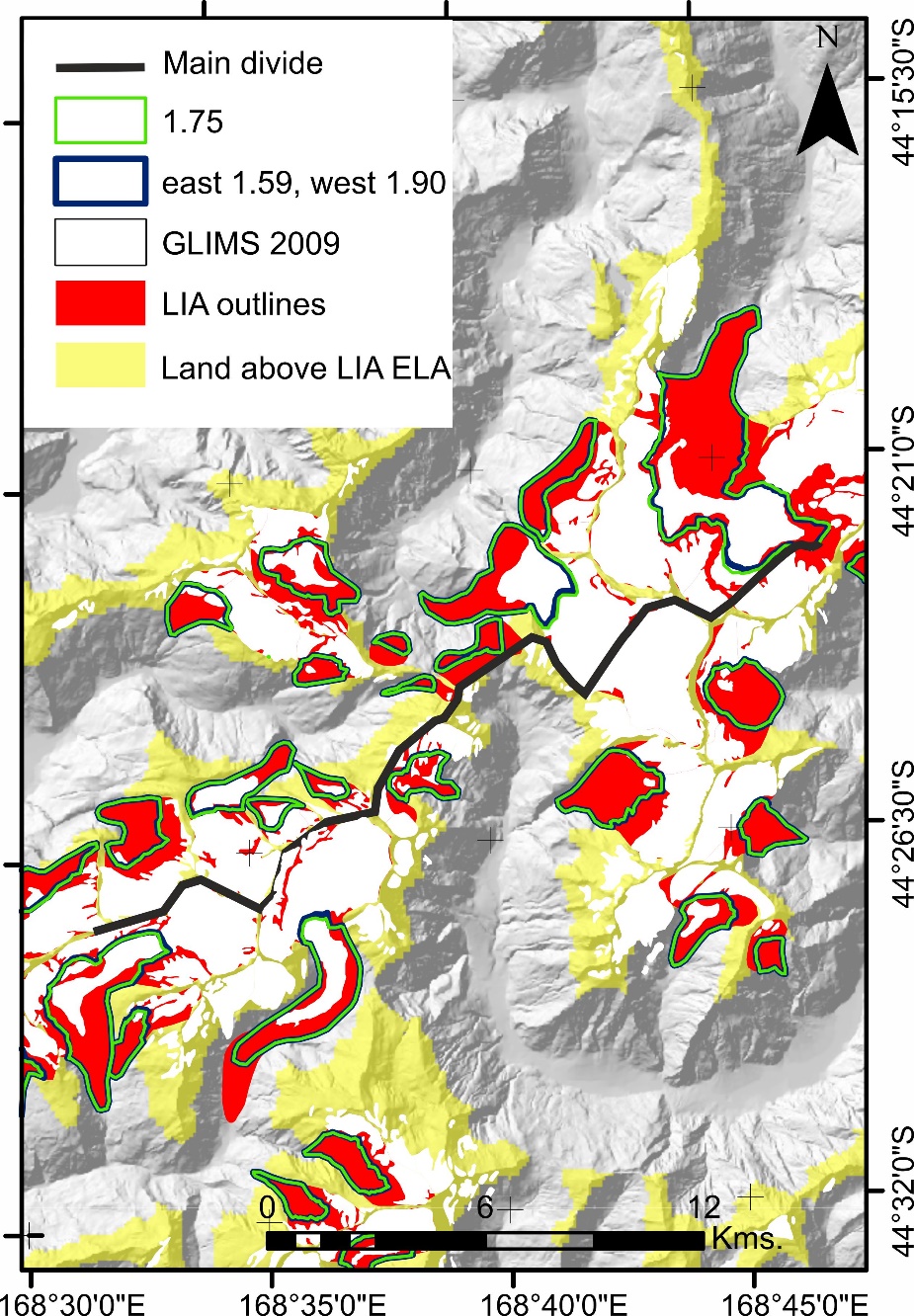


**Figure SI 2.** Example for Mt Aspiring region of land computed to be above a linear trend surface of the LIA ELA and that used to guide where to search for geomorphological evidence of LIA glacier extent. Additionally, visual comparison of two different AABR scenarios (one where all glaciers have a balance ratio of 1.75, and one where west coast glaciers have BR = 1.9 and east coast glaciers have BR = 1.59) for determining LIA glacier-specific ELA.


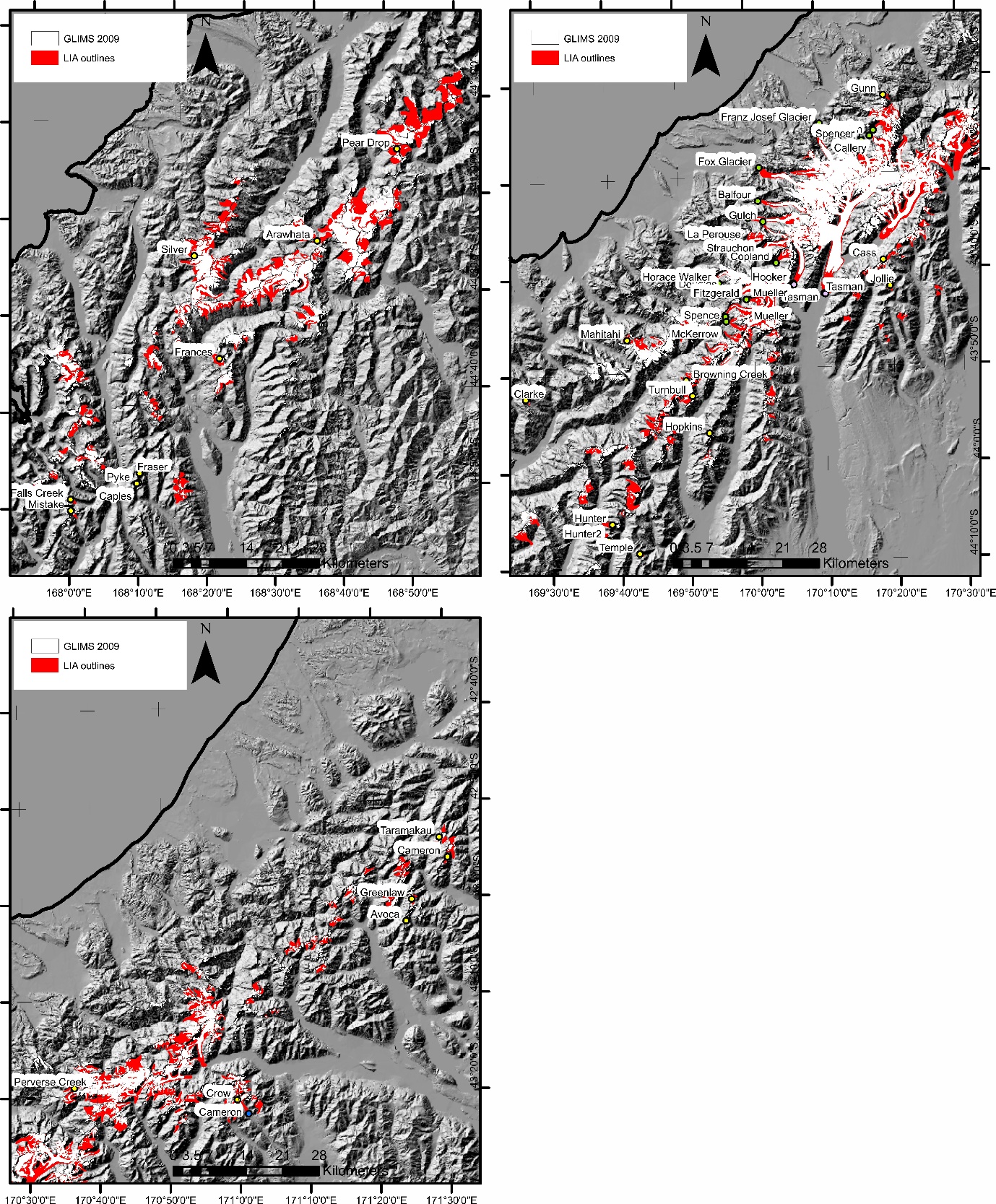


**Figure SI 3.** Little Ice Age (LIA) extent of glaciers across the Southern Alps, New Zealand.


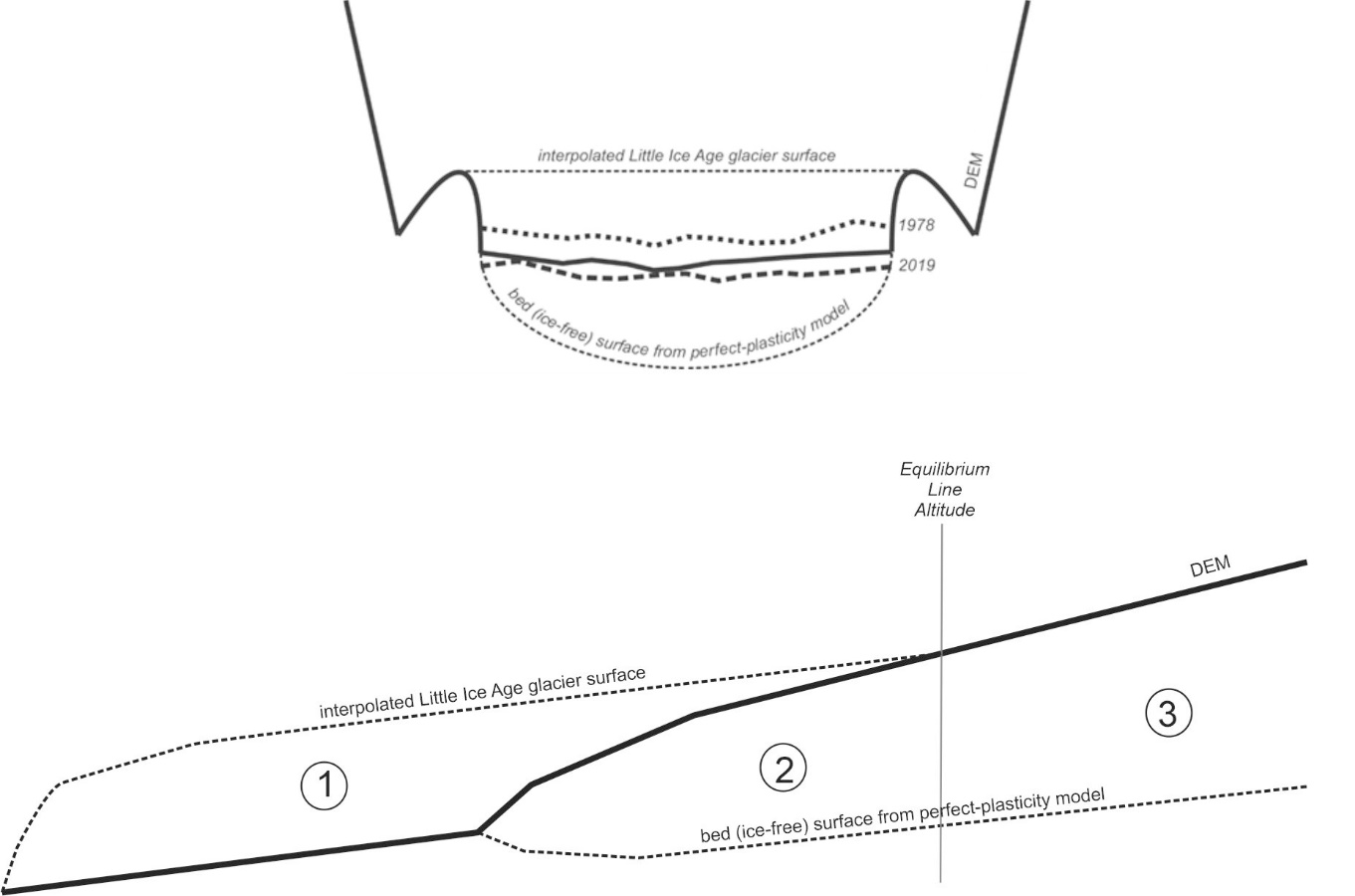


**Figure SI 4.** Sketch to illustrate method developed and used in this study to estimate ice thickness and hence volume for glaciers from outlines at different time periods. The digitised glacier outlines are draped onto a DEM to extract grid cell values at glacier margins, a surface is interpolated between those points and then the thickness is estimated by differencing that interpolated ice surface with a bed (ice-free) surface. In this study the bed (ice-free) was derived using a perfect-plasticity model (James and Carrivick, 2016). So, our LIA absolute volume is the sum of parts 1 and 2 and 3. Part 1 is the volume between LIA surface and modern DEM. Part 2 is the ice at the time of the DEM within the LIA ablation area (i.e. below the LIA ELA). Part 3 is ice at the time of the DEM above the LIA ELA.


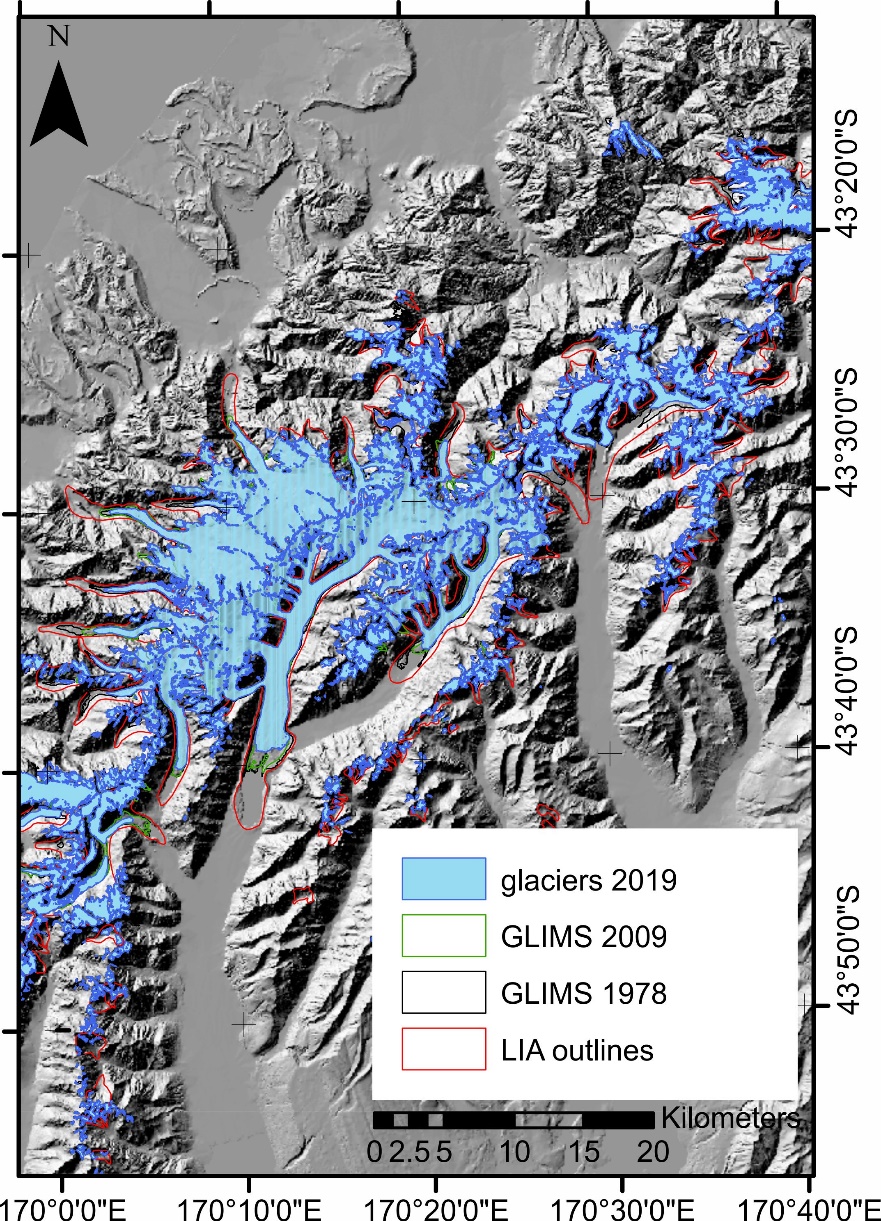


**Figure SI 5.** Example for Mt Cook and Godley regions of glacier outlines for glaciers during the LIA, 1978, 2009 and 2019.


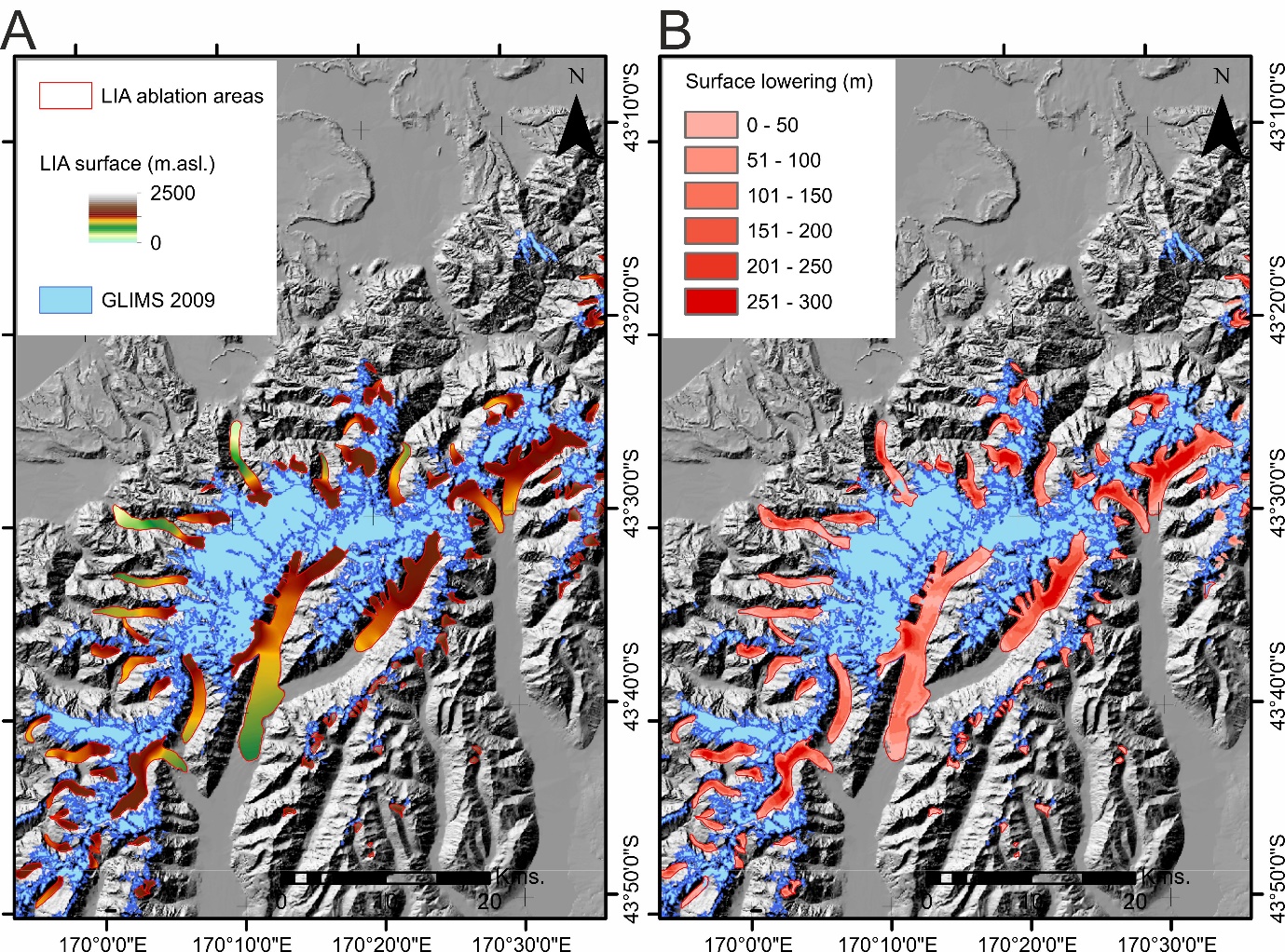


**Figure SI 6.** Example for Mt Cook and Godley regions of ice surface in LIA glacier ablation areas (A) reconstructed using a Natural Neighbour interpolation between moraine crests and the LIA ELA, where the latter was calculated using an automated AABR analysis. Thus the difference between this surface and the contemporary DEM represents surface lowering (B) and that lowering trends to zero at the ELA.


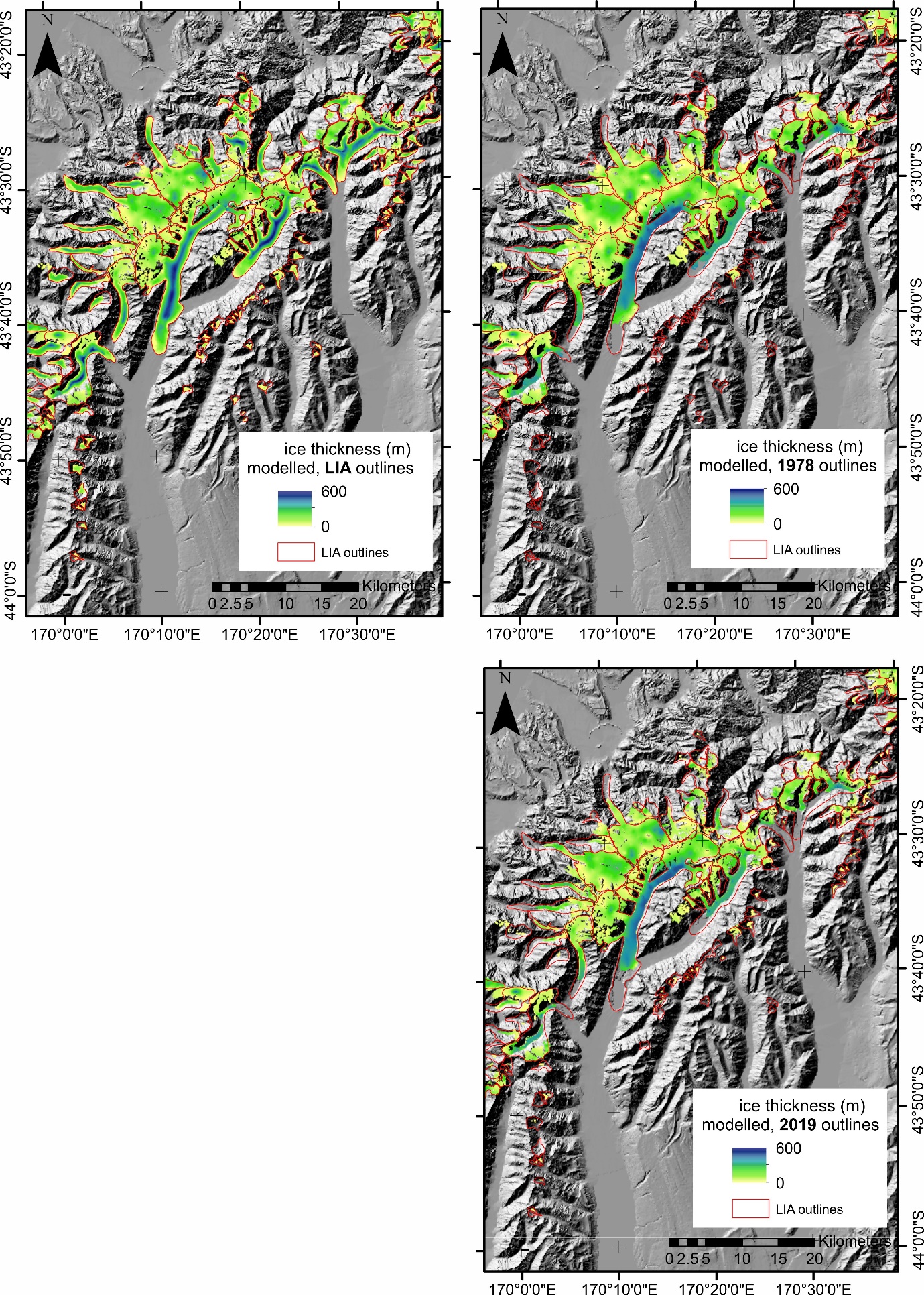


**Figure SI 7.** Example for Mt Cook and Godley regions of ice thickness grids for accumulation and ablation areas of LIA, 1978 and 2019 glaciers.

**Additional Supporting Information (Files uploaded separately)**

- **Data Set S1.** Our LIA glacier outlines and ablation areas, and our historical glacier outlines (1978, 2009, 2019) are made freely available as ArcGIS shapefiles in NZGD2000 NZTM projection.
- **Data Set S2.** Our LIA glacier ablation area surface elevation and our contemporary ice thickness maps are made freely available as ArcGIS raster grids in NZGD2000 NZTM projection.
